# Investigating active area dependent high performing photoresponse through thin films of Weyl Semimetal WTe_2_

**DOI:** 10.1038/s41598-022-27200-z

**Published:** 2023-01-05

**Authors:** Sahil Verma, Reena Yadav, Animesh Pandey, Mandeep Kaur, Sudhir Husale

**Affiliations:** 1 Academy of Scientific and Innovative Research (AcSIR), Ghaziabad, 201002 India; 2 National Physical Laboratory, Council of Scientific and Industrial Research, Dr. K S Krishnan Road, New Delhi, 110012 India

**Keywords:** Materials for devices, Materials for optics, Electronic devices

## Abstract

WTe_2_ is one of the wonder layered materials, displays interesting overlapping of electron–hole pairs, opening of the surface bandgap, anisotropy in its crystal structure and very much sought appealing material for room temperature broadband photodection applications. Here we report the photoresponse of WTe_2_ thin films and microchannel devices fabricated on silicon nitride substrates. A clear sharp rise in photocurrent observed under the illumination of visible (532 nm) and NIR wavelengths (1064 nm). The observed phoresponse is very convincing and repetitive for ON /OFF cycles of laser light illumination. The channel length dependence of photocurrent is noticed for few hundred nanometers to micrometers. The photocurrent, rise & decay times, responsivity and detectivity are studied using different channel lengths. Strikingly microchannel gives few orders of greater responsivity compared to larger active area investigated here. The responsivity and detectivity are observed as large as 29 A/W and 3.6 × 10^8^ Jones respectively. The high performing photodetection properties indicate that WTe_2_ can be used as a broad band material for future optoelectronic applications.

## Introduction

There is a great demand for photon detecting materials that are sensitive under ultraviolet, visible, infrared and especially terahertz radiations where it should be capable of detecting low energy photons preferably at room temperature. Since inception of the wonder material graphene, many new novel 2D, 3D materials including transition metal dichalcogenides have been explored for their extraordinary mechanical, electronic and optical properties^1,2^. Along this line, new class of materials such as topological insulators, Weyl semimetals show robust and distinct exotic properties due to strong spin orbit coupling and interesting metallic surface states. These materials are more attractive due to their potential applications in spintronics, thermoelectrics, energy harvesting and favourable photodetection /optoelectronic applications from UV to IR that include imaging, optical communications, sensors, wearable devices, environmental monitoring etc. Recently more attention is given to semimetals due to their terahertz or low energy photon detection capabilities which is highly sought for room temperature applications^3^.

Weyl semimetal WTe_2_ possesses a very interesting anisotropic orthorhombic crystal, band structure, overlapped electron hole pockets and exhibits perfect electron hole charge compensation. Light absorption in Weyl semimetal doesn’t depend on the bandgap of the material. The presence of Dirac linear dispersion and zero energy gap enable the detection of low energy photon down to infrared or terahertz regime. A very high non saturating magnetoresistance in WTe_2_^4^, observation of nonlinear Hall voltage in absence of a magnetic field^5^, gate tunable superconductivity^6^ etc. are some of the very exciting results reported already. Recently WTe_2_ is explored for optoelectronics applications such as polarization sensitive visible to IR detection^7^, circular photogalvanic effects^8^, generation of a picosecond spin-photocurrent^9^. Dramatic phase change in the WTe_2_ crystal structure was observed when Raman scattering spectra was studied for monolayers and few layers^10^. The thin films of WTe_2_ in combination with MoTe_2_ exhibit a large photocurrent enhancement with self-powered approach without need of any bias voltage and effectively overcome the problem of dark current^11^. Recent theoretical study reports that photocurrent travels opposite directions compared to bulk response and surface state contributions is much more^12^. The broadband photodetection is very exciting in WTe_2_ which in principle can overcome the problems faced by other 2D materials where the detection (visible to far infrared) and polarization sensitivity are limited by their semiconductor nature and inplane symmetry, respectively. Theoretical work predicted that due to tilts in linear dispersion at Weyl points and broken inversion symmetry, WTe_2_ shows quantized circular photogalvanic effect^13^ and detection of mid or far-infrared radiations is also possible^14^.

Nanosheets, microcrystals of WTe_2_ have been successfully studied for broad spectral/terahertz photodetection^7,15^. Compared to nanostructures, thin films of WTe_2_ are better for technological applications due to less complexity in device fabrication but the optoelectronics properties of sputtered WTe_2_ thin films and their nanoscale active area dependent photodetection remain relatively unexplored. Here we report photorepsonse study of sputtered deposited WTe_2_ thin films that have been thoroughly characterized to confirm their crystalline quality. Since semiconductor technology based applications have moved towards the use of nanodevices, it is important to investigate active area dependent photoresponse of material. The dependence of photoresponse on device’s channel length has been systematically studied under the illumination of laser light irradiations. Further, we report the variation of photocurrent with applied bias voltage and also with the power of the incident laser at room temperature. Results are analysed in two sections, the photoconductivity studied for a specific area is presented first and photodetection study over a device active area is shown in the latter part.

## Experimental

Thin films of WTe_2_ were deposited using DC sputtering machine (Excel Instruments) on Si_3_N_4_/Si (Silicon of about 500 µm and on top LPCVD grown nitride of about 100 nm) and substrates were pre-cleaned in a sequential manner by using acetone, isopropanol, methanol and deionized water. Thickness of the film is characterized by performing cross sectional SEM images and height profilometer. Thin films are also characterized by using EDS (Zeiss Auriga), UV absorption and Raman spectroscopies to know the elemental composition, estimation of bandgap of the material and crystalline quality of the material respectively. Further to carry out optoelectronic measurements, gold metal pads were deposited on the WTe_2_ films using a shadow mask technique and microchannels were formed by depositing the metal electrodes using FIB based metal deposition technique (gas injection system, Zeiss Auriga). Note that thin films were directly deposited on the substrate using shadow mask where the active device area was lower than the electrode dimensions and experimental conditions are shown in the supplementary material table I. Probe station (Cascade Microtech EPS150TRIAX) equipped with shield enclosure (EPS-ACC-SE750), source meter 2634B and laser light sources were used to measure the electrical response (source –drain IV characteristics) of the samples in presence and absence of laser light irradiations.

## Results and discussion

**Figure 1:**
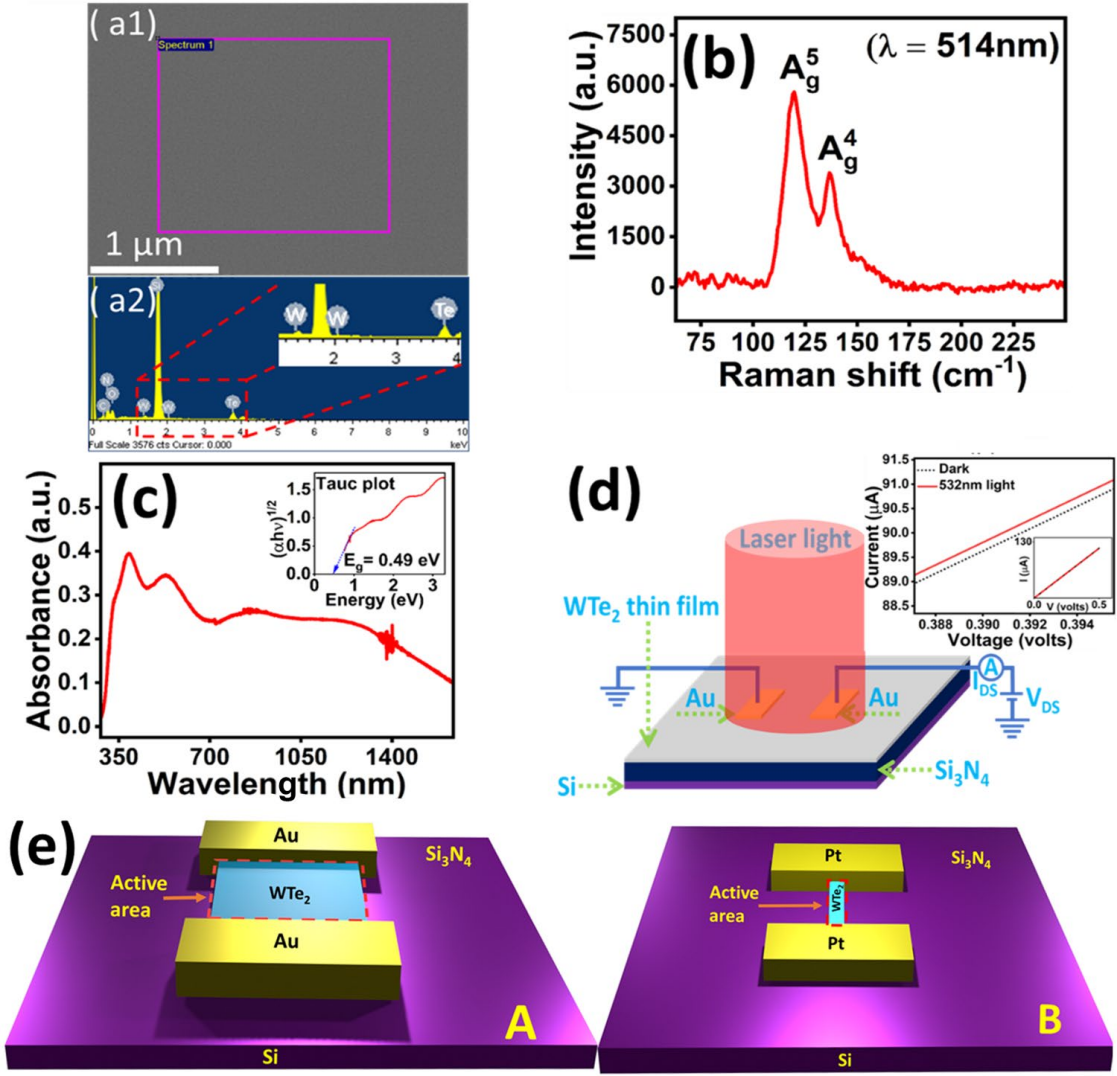
Deposition and characterization of WTe_2_ films : (**a1**) Shows FESEM image along with EDS spectra (**a2**) of the selected portion. (**b**) Represents Raman spectra of the film. (**c**) Shows the UV absorption spectra and estimation of bandgap (inset). (**d**) Schematics of the optoelectronic measurements and curve in the inset represents IV characteristics of the film. (**e**) Different device configurations used in this study. Deposition of WTe_2_ films using shadow masks and gold pads (A). FIB etched WTe_2_ films and Pt contacts (B).

Figure 1(a1) shows the FESEM image of the sputter deposited WTe_2_ film which is very uniform without any noticeable grains indicating the smooth surface. The pink square represents the area used for energy dispersive X-ray spectroscopy (EDS) analysis of the deposited film to know the elemental composition of the material. The EDS spectra (Fig. 1a2) shows the presence of tungsten and telluride peaks with the atomic weight percentage was observed very close to the atomic weight ratio percentage 1:2 (supplementary material table II).

The quality of the material was investigated using Raman spectroscopy (Fig. 1b). The Raman spectra shows two peaks which corresponds to the *A*^*5*^_*g*_ and *A*^*4*^_*g*_ vibrational modes and values are very close to earlier reported vibration modes centered at (119.7 and 136.9 cm^-1^) and experimental findings obtained using polarized Raman scattering studies^10^. To estimate the bandgap of the material UV absorption spectra (Fig. 1c) was carried out. The spectra shows wide range absorption that indicates WTe_2_ is a very good light sensing material. The band gap of about ~ 0.49 eV was estimated through Tauc plot as shown in the inset Fig. 1c. Figure 1d shows the device schematics used to study the photoresponse of WTe_2_ films. For electrical transport measurements, the metal pads were deposited with the help of shadow mask in the sputtering machine whereas microchannel devices were fabricated using GIS (gas injection system) present in FIB system as discussed in our earlier works and elsewhere^16,17^. Laser light irradiation on the sample was fully covering the electrodes because laser spot diameter was greater than the size of the photodetecting active area including the metal electrodes. Inset in Fig. 1d shows the IV characteristics of the film which is linear in nature representing a good ohmic contact. Figure 1e represents the different device geometries used in this study to change the active area of the device. Data obtained using device geometries Fig. 1e(A) and Fig. 1e(B) are reported in this manuscript.

**Figure 2:**
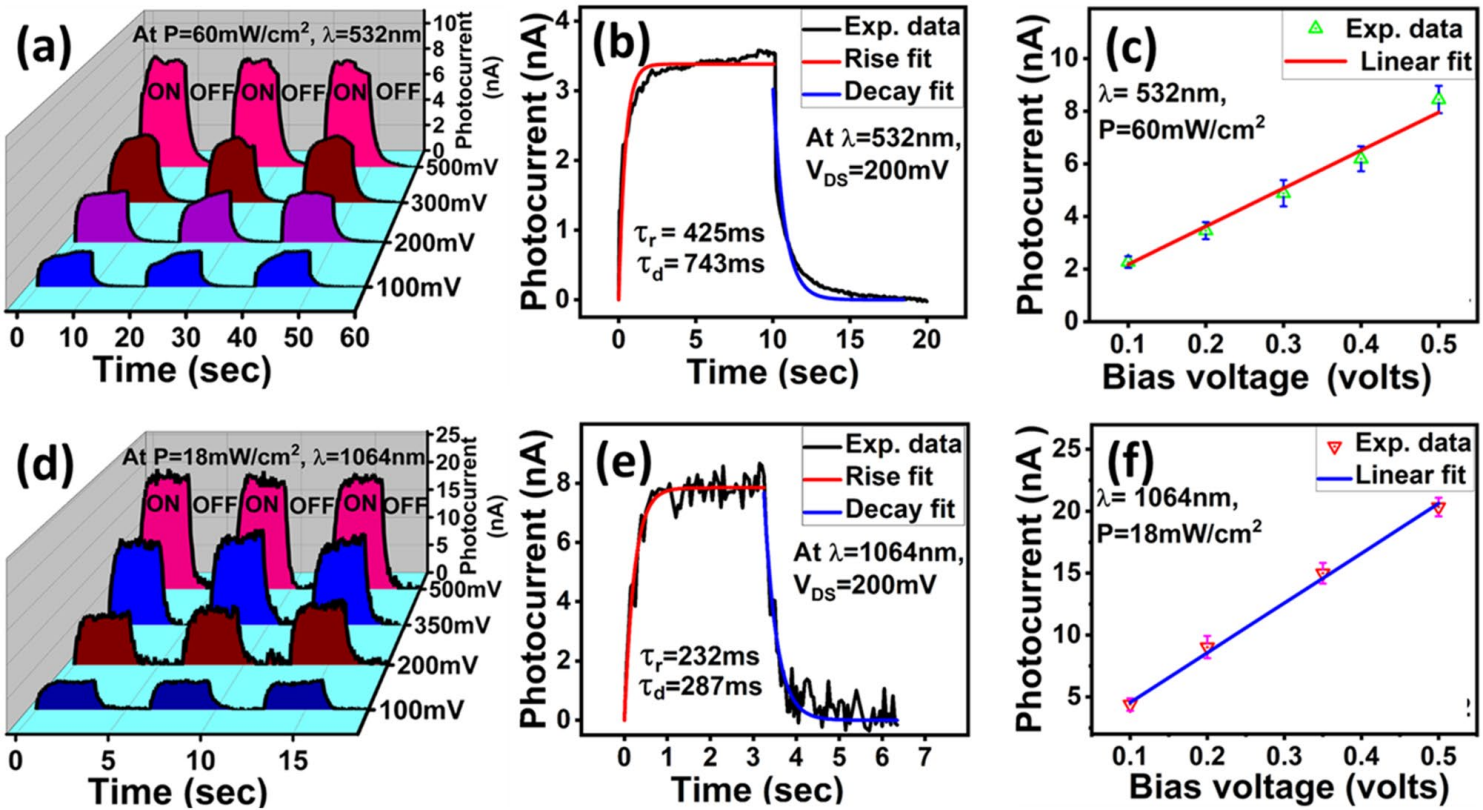
Time dependent photocurrent measurements. (**a**,**d**) photoresponse of WTe_2_ films under 532 and 1064 nm laser irradiations respectively. (**b**,**e**) Red and blue curves represent rise and decay fit constants. (**c**,**f**) Bias dependent photocurrent measurements under the illumination of laser lights 532 and 1064 nm respectively.

Photoresponse of these fabricated devices were systematically studied for the various devices reported here. The longer channel length (⁓550 µm) film was deposited using a shadow mask technique whereas devices with channel lengths ≤ 15 µm have been fabricated using a focused ion beam milling method. Figure 2a shows the time dependent photocurrent measurements performed at different bias voltages. The ‘ON’ as shown in the Fig. 2a indicates the laser light is ON and active device area is exposed due to the light illumination. The laser light was switched ON and OFF for every 10 secs interval time. A clear and detectable change in device’s current was observed. The photocurrent *I*_*ph*_ was estimated using the relation

$$I_{ph} = I_{light} - I_{dark }$$

where *I*_*light*_ and *I*_*dark*_ are the currents measured when light was switched ON and OFF respectively.

Since the generation of photocurrent depends on the incident power of the laser, quantum efficiency of the material and wavelength or energy of the photon, the increase in photocurrent may also be understood from the relation

$$I_{{ph}}\propto \eta P/hv$$

where *P* is the incident power, *hv* is the photon energy, *η* is the quantum efficiency i.e. photon generated carrier density.

During bias dependent photocurrent measurements, power of the laser light was kept constant and measurements were repeated for bias voltages 100, 200, 300 and 500 mV as shown in the Fig. 2a,d. The rise and decay time constants are estimated using the following relations. $I_{ph} = I_{o } - I_{o} \left( {e^{{\frac{ - x}{{\tau_{r} }}}} } \right)$ and $I_{ph} = I_{o} + A_{1} e^{{\frac{ - x}{{\tau_{d} }}}}$ respectively. Where $I_{o}$= saturated value of the photocurrent, $\tau_{r}$= rise time constant, $\tau_{d}$= decay time constant and $A_{1}$ is the fitting parameter.

The fitting curves give rise (red curve) and decay (blue curve) time constants of about 420 ms and 740 ms respectively (Fig. 2b) when the sample was illuminated with the visible laser 532 nm. Figure 2c shows that as the applied bias voltage was increased the photocurrent was also increased indicating the photocurrent dependence on the applied bias voltage and WTe_2_ material responds well under different applied bias voltages. The time dependent photocurrent measurements were repeated under the illumination of NIR wavelength (1064 nm) and the similar trend of clear rise and decay in photocurrent was observed when light was switched ON and OFF respectively (Fig. 2d). The estimated rise and decay time constants (~ 230 and 290ms, Fig. 2e) are found to be better as compared to response under visible light (Fig. 2b). Earlier reports also indicate that Weyl semimetals show broadband response and demonstrated that low energy photons (infrared wavelengths) effectively excite electrons in the lower part of the Weyl cone to the upper part^18,19^.

Here we have extensively studied the device’s photocurrent in presence of different applied bias voltages at a fixed incident power of the laser beam and again measurements were repeated for different power. The increase in photocurrent value was observed as we increase the bias voltage. The curves are shown in Fig. 2c,f. The systematic linear increase in the photocurrent is clearly visible from the curves as shown in the figures. The bias dependent increase in photocurrent has been studied previously for other topological, semimetals and 2D materials which is consistent with our results^20,21^. At high bias, it is expected to have reduction in transit time and increase in the drift velocity which can be explained by the following equation.

$\tau_{t} = l^{2} /\mu V$ where *V* is a bias voltage, *l* is the channel length and µ is the carrier mobility.

**Figure 3:**
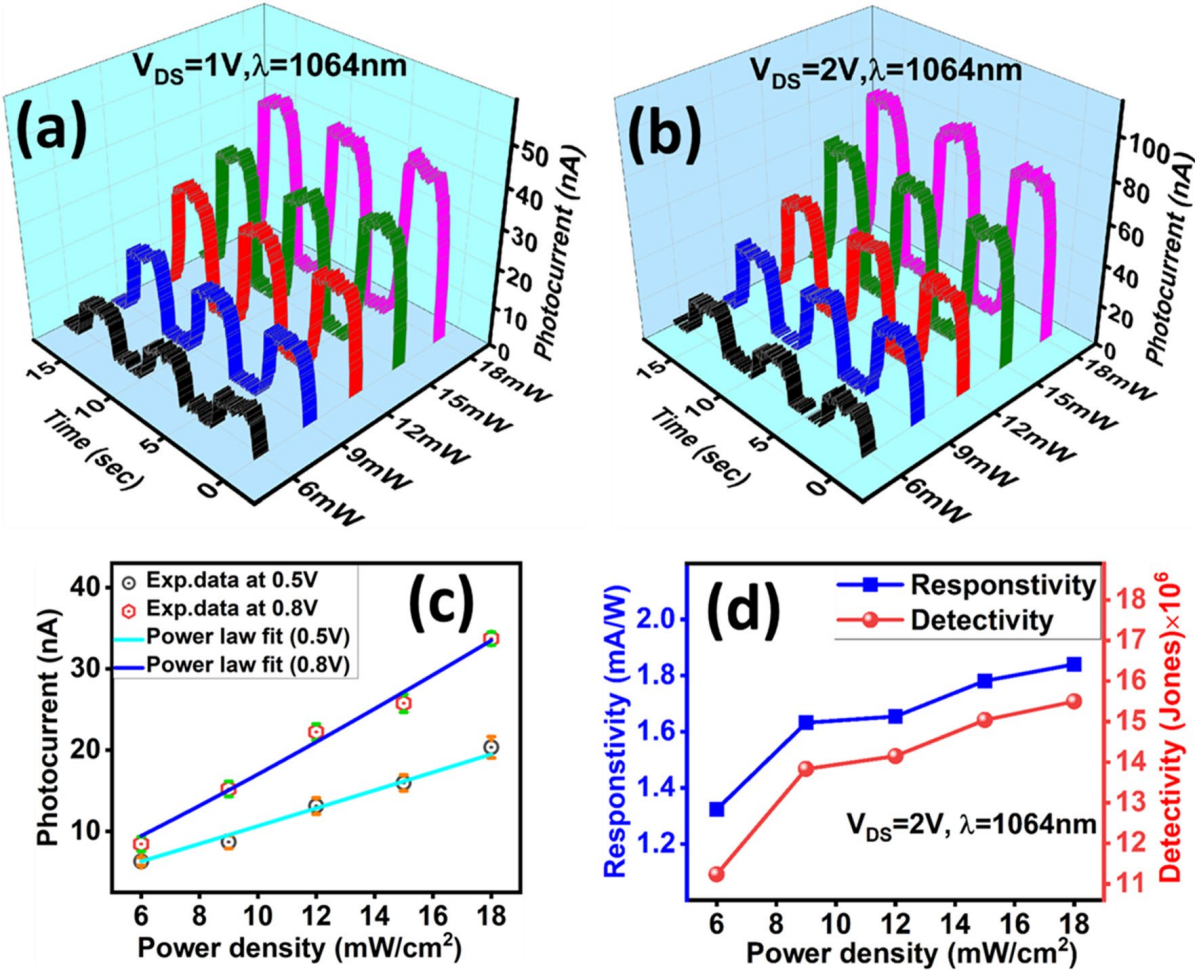
power dependent photocurrent measurements at different bias voltages 1 V (**a**), 2 V (**b**) and 0.5 V& 0.8 V (**c**). Blue and cyan line curves represent power law fit (**c**). (**d**) shows responsivity (blue curve) and detectivity (red curve) measured at bias voltage 2 V.

Figure 3 shows the laser power (1064 nm) dependent photocurrent measurements carried out at 1 V (Fig. 3a) and 2 V (Fig. 3b). The power of the laser was varied from 6 to 18 mW and corresponding change in the value of current was measured. The increase and decrease in photocurrent corresponds to light ON state and OFF state carried out for every  3s’s interval time. A noticeable rise in the magnitude of photocurrent due to the increase in laser power is clearly visible for both bias voltages as shown in Fig. 3a,b. This power dependence data is convincing to indicate that WTe_2_ is nicely responding to increase in photon irradiation. To know the relation between change in power density and observed photocurrent, we plot the power density as a function of magnitude of photocurrent for different bias voltages as shown in the Fig. 3c. Here we used a power law relation i.e. I_ph_ ⁓ P^θ^ and fit the power dependent photocurrent data obtained for the 0.5 and 0.8 voltages. Fit shows the linear behavior with the exponent value close to 1. This indicates that observed photocurrent has minimal effects from other sources like thermoelectric currents. Recent work on WTe_2_ also reports power dependent photocurrent measurements and linear response was observed^7^.

For any photodetector, responsivity (R) and detectivity (D) are very important parameters which we have estimated using following relation.

$R_{ph} = \frac{{I_{ph} }}{{P_{d} \times A}}$, $D = \frac{{R_{ph} \sqrt A }}{{\sqrt {2qI_{D} } }}$ where *A* is the active device area, *P*_*d*_ is the incident power density I_D_ -dark current , q- is the electrons charge and *I*_*ph*_ is the photocurrent measured at certain bias voltage.

Figure 3d shows the estimated values of responsivity and detectivity plotted as a function of laser power density for channel length of about 550 µm. Since responsivity depends on the active area, the carrier transfer time and lifetime of the photo-induced carrier. Hence responsivity can be expressed as $R \propto \frac{{\tau_{lifetime} }}{{\tau_{transit} }}$.

**Figure 4:**
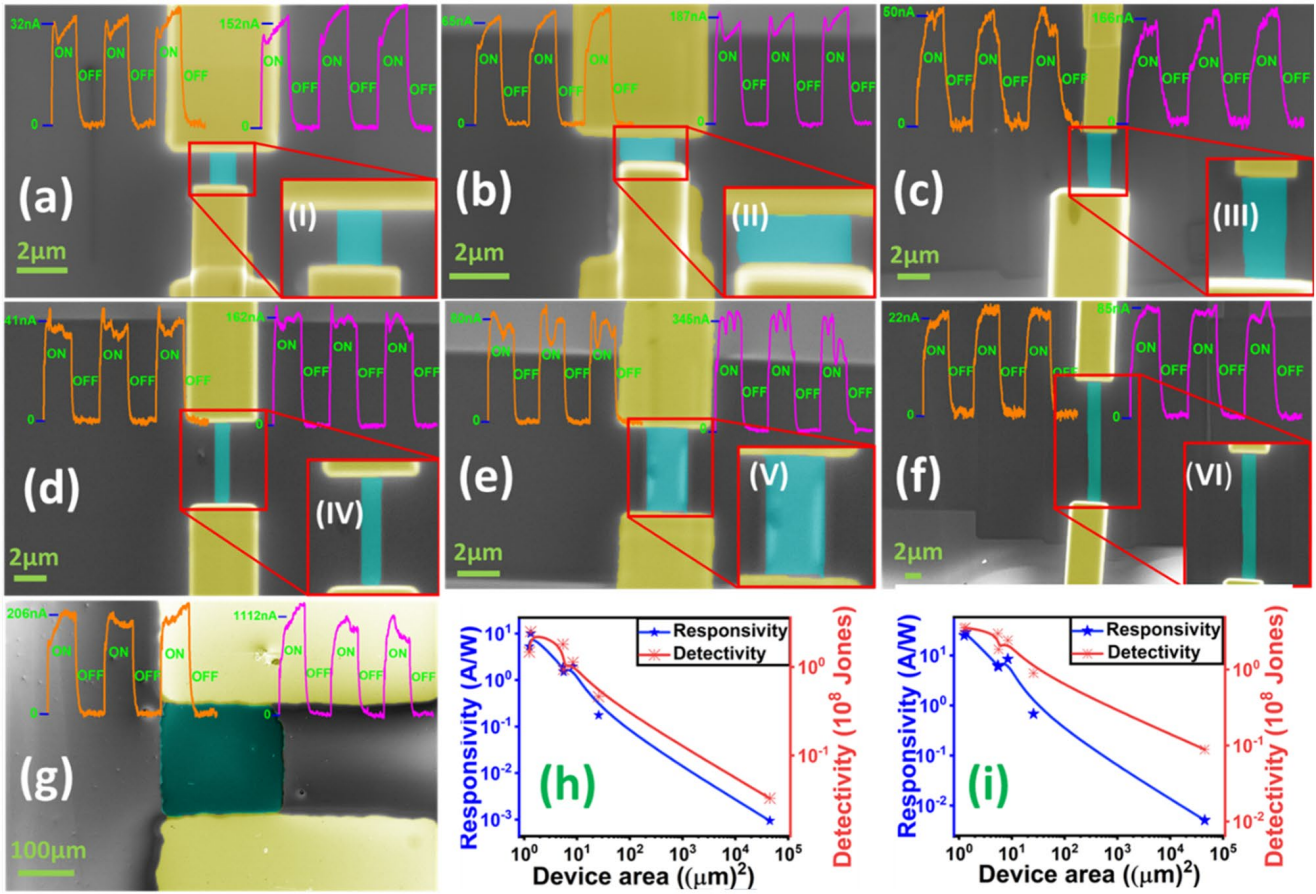
Active area dependent photocurrent measurements. (**a**–**g**) False coloured FESEM images of different active areas and insets (I- VI) show the zoom section of constricted WTe_2_ channel respectively. Curves (orange and magenta) on top of FESEM images represent time dependent photocurrent measurements performed at bias voltages 0.5 V and 2 V respectively. (**h**,**i**) Show the responsivity (blue) and detectivity (red) curves at bias volt 0.5 V and 2 V respectively.

To investigate the dependence of responsivity on the active area, we fabricated devices by using shadow mask and FIB etching technique. False coloured FESEM images of the devices are shown in the Figs. 4a–g and improvement in device’s responsivity was thoroughly studied. Surprisingly very good photoresponse was observed which is shown in the Fig. 4a-g (orange and magenta curves). Color curves shown on top of FESEM images represent the device’s response under the illumination of laser light 532 nm. A clear change in device’s photocurrent was observed for all the active areas studied here. The increase in responsivity was noticed with decrease in the channel length. Note that, the micro channels were made with Pt as metal electrodes using GIS (FIB attachment for metal deposition). The photocurrent measurements have been carried out for different bias voltages and repeated for seven different active areas (supplementary table I) and data of responsivity & detectivity at bias voltages (0.5 and 2 V) are shown in the Fig. 4 (h and i) respectively. The rapid increase in responsivity was noticed with decrease in size of active area which is consistent with the SnTe photodetector^22^ where responsivity was observed proportional to 1/L^2^.

Our results indicate that use of 1.17 µm channel compared to 220 µm channel length exhibits ~ 4 orders improvement in the responsivity and ∼ 3 × improvement when responsivity was compared with the 4 µm channel length. This could be due to decrease in active area which also reduces the possibility of having defects or less grains and grain boundaries and the photoconductivity depends on these parameters also. The enhancement in the responsivity can be understood mathematically from the relation *R* = *I*_*ph*_*/AP*_*d*_ where A is the active or effective area calculated from the length x width between the two electrodes and P_d_ is the power density. This tells that responsivity depends on both effective area and power of the laser. The area of shadow mask deposited channel is about 205 × 220 um^2^ and microchannel of about 1.17 × 1.07 µm^2^ which estimates more than four orders of change in the device area. Further the ratio of carrier lifetime to transit time represents the gain of the device i.e. $\Gamma =\frac{\tau }{{\tau }_{t} }$. This indicates that the ratio of $V/{l}^{2}$ of the microchannel is several orders larger than shadow mask channel device that gives shorter transport time of carrier and thus provides a greater probability of carrier collection. Similarly the earlier work done on nanoflakes and bulk samples of molybdenum disulfide (MoS_2_) showed several orders of enhancement in responsivity due to the change in active device area which was six orders of magnitude more compared to the bulk sample^23^. It is important to note that sputtered thin films may not be that uniform over a large area and hence small active areas may be better. Decrease in channel length corresponds to decrease in transit time which could increase the mobility. The channel dependent photocurrent studies on MoS_2_ flakes observed that smaller channel length shows a higher responsivity^24^. The work on black phosphorus also reported two orders of change in repsonsivity values when the channel length was decreased from 1 µm to 100 nm due to the smaller transit time and larger transverse electric field^25^. All these reports indicate that responsivity can be enhanced further with the scaling of the channel length.

Recently Weyl semimetal material have triggered many theoretical investigations on photocurrent study and all of them pointed out that the presence of type –II Dirac fermions enables the strong broad band light absorption through these materials. Due to the breaking of Lorentz invariance (crystalline symmetry), these Weyl fermions forms tilted cones around the Fermi level compared with other Dirac materials which make them more efficient for the generation of electron hole pairs. Supplementary Fig. 1 shows the hypothetical predication of photocurrent generation in type II Weyl semimetal based on the fact that the Weyl cones in WTe_2_ are strongly tilted and the Fermi surface crosses both valence band and conduction band^26^. This results in an unstable photoexcited carriers in the conduction band and electrons from valence band to conduction band can transist without any need of external bias. Also it is expected to have higher carrier mobility, robust Fermi arc type surface states supporting the greater photocurrent efficiency. The laser light exposure excites the electrons from valence band to conduction band which might cause the band renormalization and proposed breaking of inversion symmetry ending up in a noncentrosymmetric Weyl materials. Published reports indicate that noncentrosymmetric Weyl materials can be advantageously applied to room temperature detections of mid- and far-infrared radiations^27^. Here our results demonstrate the best responsivity of about 29 A/W for channel length of about 1.17 µm which we find very competitive when compared with the published literature as shown in Table 1. Mainly the semimetals or Weyl materials are shown in the following Table 1.

**Table 1:** Semimetals or Weyl materials based photodetectors.

Sr. No	Material	Wavelength	Responstivity (mA/W)	Detectivity (Jones)	Ref
1	TaAs	438.5 nm	179 × 10^–3^	4.5 × 10^7^	^28^
		2.82 µm	78 × 10^–3^	1.88 × 10^7^	
		10.29 µm	88 × 10^–3^	2.34 × 10^7^	
2	MoTe_2_	532 nm	0.40	1.07 × 10^8^	^29^
		10.6 µm	4.15 × 10^–2^	9.1 × 10^6^	
3	TaIrTe_4_	633 nm	0.34	2.7 × 10^7^	^30^
		4 µm	30.2 × 10^–3^	2.5 × 10^6^	
		10.6 µm	20 × 10^–3^	1.4 × 10^6^	
4	SnTe	940 nm	1710	3.46 × 10^11^	^31^
5	MoTe_2_-graphene	1300 nm	200		^32^
6	Graphene quantum dot	800 nm	500	-	^33^
		400 nm	200	1.1 × 10^11^	
7	PtTe2	633 nm	0.034	–	^34^
8	SmB6	10.6 µm	1.99	2.5 × 10^7^	^35^
9	ZrTe5	632 nm	4.5	–	^36^
10	Cd3As2	633 nm	5.88	–	^37^
11	WTe_2_	532 nm	29 × 10^3^	3.6 × 10^8^	This work

Our work indicate that short channel lengths based devices may be more relevant to study photocurrent response under low energy photon radiations (THz). Recently highly efficient THz photoresponse was detected in PdSe_2_ flakes where 500 nm channel length and bow- tie antennas structures were used to observe the THz radiation into the localized oscillating electric field^38^. This work suggests that nanowires and nanoribbons of WTe_2_ can be further studied for achieving high performance properties using length dependence approach.

## Conclusions

Here we have studied broad spectral photoresponse of WTe_2_ thin films under the illumination visible and NIR wavelengths at room temperature. The photoresponse was also studied for different channel lengths from few hundreds of microns down to few hundreds of nm. We observed that devices with short channels show better photoresponse compared to longer channel length devices. The clear noticeable photocurrent was measured with fast rise and decay times. The stability of photocurrent value was noticed for repetitive light ON/OFF cycles. The best responsivity 29 A/W and detectivity 3.6 × 10^8^ Jones were observed for the channel length of 1.17 µm which indicates that devices prepared by using WTe_2_ will be high performing. Overall our results, first time report the photoresponse studies on sputtered deposited WTe_2_ films and represents robust nature of this material for photocurrent measurements at room temperature. Further indicate that WTe_2_ can be used in optoelectronic devices for broadband applications and may simulate more work in the future for potential applications in the detection of low energy photons, optical communications, THz absorbers / sensors, security scanning etc.

## Supplementary Information

Supplementary Information.

## Data Availability

All of the data supporting this work will be made available from the corresponding author upon reasonable request.
