# Supplementary material for: Investigating active area dependent high performing photoresponse through thin films of Weyl Semimetal WTe2
Source: Sci Rep. 2023 Jan 5;13:197. doi: 10.1038/s41598-022-27200-z (PMC9814664; doi:10.1038/s41598-022-27200-z)
Supplement: Supplementary file 1 — Supplementary Information. [file 41598_2022_27200_MOESM1_ESM.pdf]

Investigating active area dependent high performing photoresponse through thin films of Weyl Semimetal WTe_2

Sahil Verma[1,2], Reena Yadav[1,2], Animesh Pandey[1,2], Mandeep Kaur[2] and Sudhir Husale[1,2,*]

[1]Academy of Scientific and Innovative Research (AcSIR), Ghaziabad-201002, India

[2] Council of Scientific and Industrial Research, National Physical Laboratory, Dr. K S Krishnan Road, New Delhi-110012, India

[*]E-mail: husalesc@nplindia.org

Supplementary table I : Experimental conditions for microchannels

Sr. No.	Length (µm)	Width (µm)	Area (µm)^2	Bias voltage (volts)	Photo Current (nA)	Dark Current (µA)	Responsivity (A/W)	Detectivity (10^8 Jones)
1	1.17	1.07	1.25	2	152	200	25	3.49
2	1.90	0.7	1.33	2	187	268	29	3.60
3	3.4	1.6	5.44	2	166	75	6.27	2.98
4	5	1.12	5.6	2	162	181	5.95	1.85
5	4.24	1.98	8.39	2	345	317	8.46	2.43
6	15.43	1.67	25.76	2	85	46	6.7×10^{-1}	8.9×10^{-1}
7	220	205	45100	2	1112	461	50.7×10^{-4}	9×10^{-2}

Supplementary table II

EDS data representing W: Te ratio

**Sr.No.**	**Sample number**	**Spectrum location**	**Atomic %age**		**Ratio W:Te**
			**W**	**Te**	
1	1	1	3.91	7.97	1 : 2.03
		2	4.21	8.50	1 : 2.01
2	2	1	2.59	5.85	1 : 2.25
		2	2.76	6.09	1 : 2.2
3	3	1	4.77	10.45	1 : 2.19
		2	5.18	10.95	1 : 2.11
4	4	1	2.39	5.65	1 : 2.36
		2	2.80	6.22	1 : 2.22
5	5	1	4.29	9.41	1 : 2.19
		2	4.85	10.09	1 : 2.08
6	6	1	4.16	8.73	1 : 2.09
		2	4.02	8.73	1 : 2.17

Supplementary Figure 1

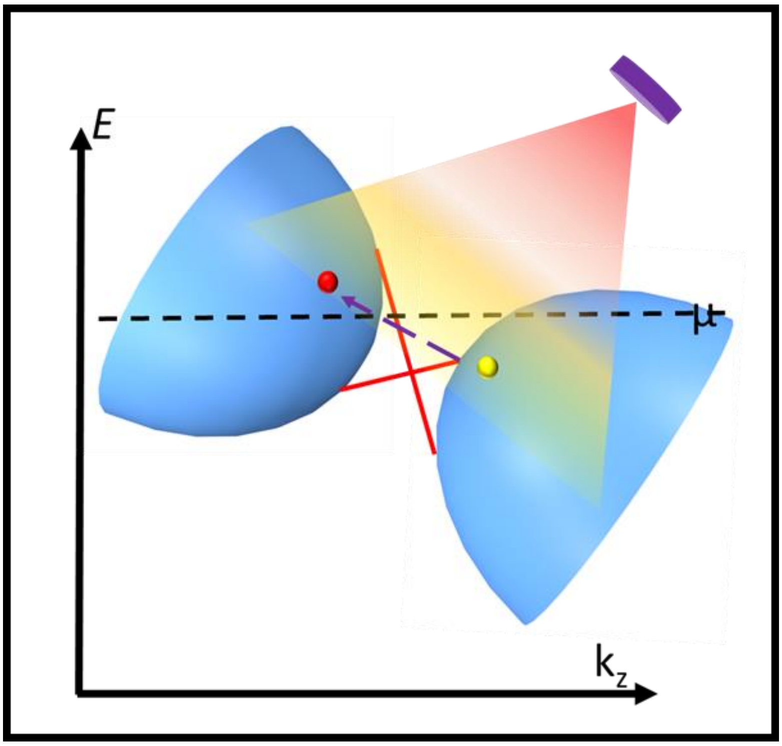

SM Fig 1 shows the schematic of photocurrent generation in Weyl semimetal.
